# New Intraoral Scanner-Based Chairside Measurement Method to Investigate the Internal Fit of Crowns: A Clinical Trial

**DOI:** 10.3390/ijerph17072182

**Published:** 2020-03-25

**Authors:** Maximiliane Amelie Schlenz, Jonas Vogler, Alexander Schmidt, Peter Rehmann, Bernd Wöstmann

**Affiliations:** Justus Liebig University, Dental Clinic - Department of Prosthodontics, Schlangenzahl 14, 35392 Giessen, Germany; jonas.a.vogler@dentist.med.uni-giessen.de (J.V.); alexander.schmidt@dentist.med.uni-giessen.de (A.S.); peter.rehmann@dentist.med.uni-giessen.de (P.R.); bernd.woestmann@dentist.med.uni-giessen.de (B.W.)

**Keywords:** CAD-CAM, internal fit, chairside, intraoral scanner, replica technique, dental crowns

## Abstract

To measure the internal fit of the computer-aided designed/computer-aided manufactured (CAD/CAM) crowns, a new scanner-based chairside approach was investigated in patients, and the results were compared to the established silicone replica technique and a digital laboratory replica method. Thirty full-coverage crown preparations were included. Based on a digital impression with an intraoral scanner (IOS, Trios 3), three CAD/CAM measurement copings (‘COM’, resin composite; ‘ZIR’, zirconium dioxide; ‘NPA’, non-precious alloy) were fabricated for each tooth preparation. The internal fit of the measurement copings was analyzed with three different evaluation methods: IOS-based digital approach (D-IOS), digital replica method with laboratory software (D-GOM), and conventional silicone replica technique (CV-SR). The congruence between the determined target parameter of the 80-µm cement space and the actual measured internal gap was investigated. Statistical analysis was performed by ANOVA (*p*-value < 0.05). No significant difference was determined between the three evaluation methods. However, significant differences were observed for the three coping materials (*p*-value < 0.05), the single measurement position (marginal, axial, and occlusal fit) (*p*-value < 0.05), and the interaction between the coping material and the measurement position (*p*-value < 0.05). COM revealed the smallest internal gap, followed by ZIR and NPA. Regardless of the coping material, the occlusal gap was higher than the axial and marginal gaps. Furthermore, only the internal gaps of the marginal area almost matched the target parameter of 80-µm for the cement space. D-IOS is effective for measuring internal fit of single crowns in different clinical settings.

## 1. Introduction

In light of the increasing digitalization of dentistry, especially with regard to intraoral scanning, the exact determination of the internal fit of fixed dental prostheses (FDPs) is of interest [1,2,3]. Misfit in the marginal area might lead to secondary caries or periodontitis, whereas, a large internal gap in the occlusal area can affect the boding and mechanical strength of ceramic restorations [1,4,5,6,7,8,9]. Besides intraoral scanning, further development in computer-aided design/computer-aided manufacturing (CAD/CAM) technologies offers a growing range of materials manufactured in various workflows [3,10,11,12]. Thus, there is a high demand for an easily applicable method for the analysis of the internal fit of FDPs. Preferably, there should be a chairside method in the dental office without the need of an elaborate laboratory setup and expert knowledge. 

Most evaluation methods described cannot be applied in patients without tooth extraction [13,14,15]. Thus, the indirect conventional silicone replica is still the most common approach in studies investigating the internal fit of FDPs in patients [16,17,18,19,20]. Though, this method is limited to two-dimensional analysis with a small number of measurement points. To overcome these limitations, three-dimensional approaches such as micro-CT [1], triple-scan technology [21], digital replica method [22,23], and optical coherence tomography [13] have been developed in recent years. However, micro-CT is not clinically applicable because of the need for radiography [1,21,24,25]. Furthermore, artifacts of metallic restorations might influence the analysis [13]. In contrast, the triple-scan technology and digital replica method are clinically applicable but require a complex experimental setup and expert skills [7,21,22,23,26]. Some studies have investigated the internal fit of FDPs using coherence tomography. This method seems to be promising in several areas of dentistry, but has limitations regarding material selection and requires a separate complex device that is not yet available for use in dentistry [13,27].

Recently, Zimmermann et al. developed a new three-dimensional approach based on an intraoral scanner (IOS) Cerec Omnicam (Dentsply Sirona, Bensheim, Germany) with the software OraCheck (Cyfex, Zurich, Switzerland) in a laboratory setup [28]. In a previous laboratory study, we systematically investigated a novel IOS-based evaluation method with the IOS Trios 3 (3Shape, Copenhagen, Denmark). The results were in good accordance compared to a digital replica method and the well-established analog silicone replica method [29]. However, in a clinical environment, saliva, various preparation geometries, or subgingival margins might affect the reliability of the approach. To the best of our knowledge, no systematic clinical evaluation has been described yet, comparing established methods with the new IOS-based approach in patients.

Hence, the aim of this clinical trial was to evaluate three different evaluation methods: the new IOS-based digital approach (D-IOS) [29], a digital replica method with laboratory software (D-GOM) [7] and the conventional silicone replica technique (CV-SR) [30]. Taking into account different CAD/CAM materials, resin composite (COM), zirconium dioxide (ZIR), and non-precious alloy (NPA) were used as measurement copings. In the present study, the following null hypothesis was tested: there is no statistically significant difference between the evaluation methods (CV-SR, D-GOM, D-IOS) or the materials (COM, ZIR, NPA) and the target parameter of 80-µm cement space matches the actual measured internal gap.

## 2. Materials and Methods 

**Figure 1:**
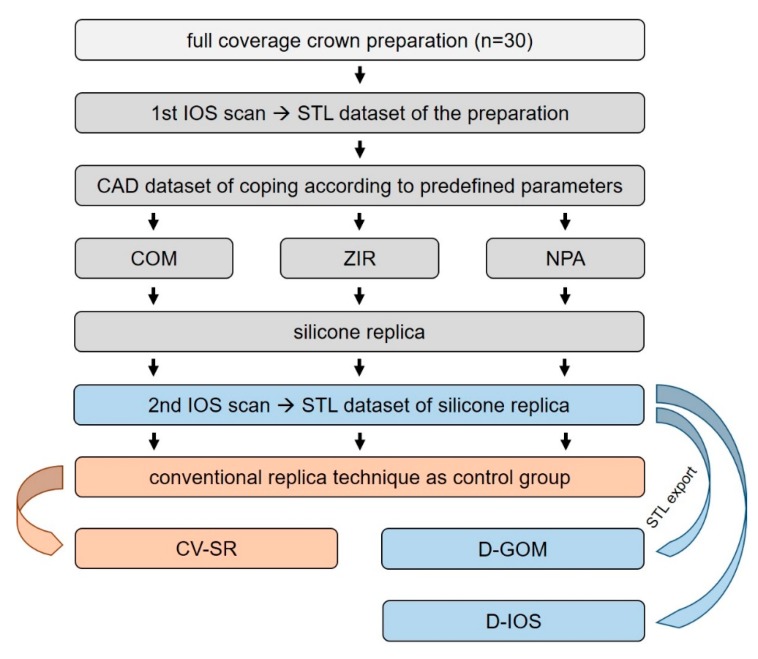
Flow scheme of the clinical trial.

Altogether 30 preparations in 20 patients (12 females, 8 males; age 35–87 years) were included in the present study. Treatment was performed at the Department of Prosthodontics of the Justus Liebig University Giessen (Germany) from April to November 2019. Only asymptomatic teeth that required a full-coverage preparation (crowns, bridges, or telescopic crowns) were included. Teeth with undistinguishable finish lines or inability to keep the preparation dry for impression taking were excluded. All subjects gave their informed consent for inclusion before they participated in the study. The study was conducted in accordance with the Declaration of Helsinki. The protocol was approved by the Ethics Committee of the Justus Liebig University Giessen (Ref. no. 267/13) and registered in the German Clinical Trial Register (DRKS00017049). One single operator (J.V.) performed all the experiments. The flow scheme in Figure 1 presents the study setup.

On the first appointment, preliminary treatment of the teeth, that is caries excavation or core-built up (OptiBond FL, Kerr, Bieberach, Germany; Rebilda LC, Voco, Cuxhaven, Germany), was performed if required. Teeth were prepared under constant water cooling with a chamfer line and round edges according to the preparation guidelines for full-coverage crowns using diamond burs [31]. For subgingival finish lines, the double-cord technique (Medi-Kord, La Maison Dentaire SA, Balzers, Lichtenstein) was used to retract the gingiva [32]. During impression taking, lips and cheeks were retracted with Optragate (Ivoclar Vivadent, Schaan, Lichtenstein). For digital impressions, the IOS Trios 3 (version 1.18.2.10, 3Shape, Copenhagen, Denmark) was used. Before usage, the IOS was calibrated with the respective calibration device [33]. Furthermore, a predefined scanning path was observed, beginning with the occlusal/oral surface and ending with the buccal surface [34]. Only the preparation and the adjacent teeth were scanned to obtain a higher accuracy of the scan data set. After digital impression taking, a provisional restoration (Luxatemp, DMG, Hamburg, Germany) was manufactured and temporarily cemented (Temp-Bond, Kerr, Biberach, Germany).

In the dental laboratory, measurement copings (representing the restorations) were designed using CAD software (version 17.2.1, dental system, 3Shape, Copenhagen, Denmark) according to the following parameters: 600-µm layer thickness [35,36], 200-µm edge reinforcement [35,36] and 80-µm cement gap [30]. Copings were milled from a blank of non-precious alloy (NPA), zirconium dioxide (ZIR), and resin composite (COM) on a 5-axis milling machine (A: Datron D5, Datron AG, Mühltal, Germany; Z: FinoCAM CA, Fino GmbH; P: FinoCAM W, Fino GmbH). Subsequently, all copings were manually polished according to the manufacturer’s instructions, and zirconia copings were additionally sintered in a calibrated dental ceramic oven (Austromat μSiC, Dekema, Freilassing, Germany) (room–800 °C, 40 min; 800–1450 °C, 65 min; 1450 °C, 120 min; 1450–800 °C, 45 min; 800–250 °C, 30 min; 250 °C–room) [29].

On the second appointment, the internal fit of the measurement copings was investigated with one conventional (CV-SR) and two digital (D-GOM and D-IOS) methods. First, the provisional restoration was removed, and the prepared tooth was carefully cleaned. Then, copings were isolated with powder spray (CEREC Optispray, Dentsply Sirona, Bensheim, Germany) to visualize the cement space [22], filled with low-viscosity addition-curing silicone blue-colored (Fit Test C&B, Voco, Cuxhaven, Germany) and seated under a constant pressure of 20 N [23,30] on the prepared tooth. Copings were carefully removed after a setting time of 10 min, and the remaining silicone replica on the prepared tooth was inspected. Before taking a second digital impression with IOS Trios 3 (capturing the silicone replica on the prepared tooth), cheeks and lips were retracted with Optragate and the teeth were gently air-dried. Subsequently, an over impression was taken for the CV-SR method. Therefore, an impression tray (Inlay Tray, Detax, Ettlingen, Germany) was filled with a high-viscosity green-colored and low-viscosity pink-colored vinyl polyether silicone to pick up the blue-colored silicone replica on the prepared tooth. After a setting time of 10 min, the impression was removed and filled with low-viscosity yellow-colored vinyl polyether silicone. This completed the trials on patients who subsequently received their regularly planned restoration. The materials used in this study are listed in Table 1. 

**Figure 2:**
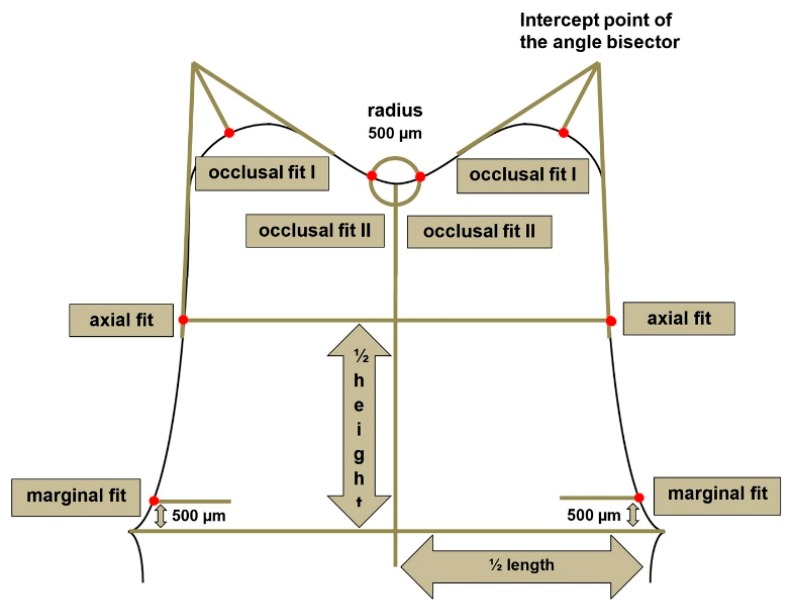
Schematic drawing of the measurement points.

**Figure 3:**
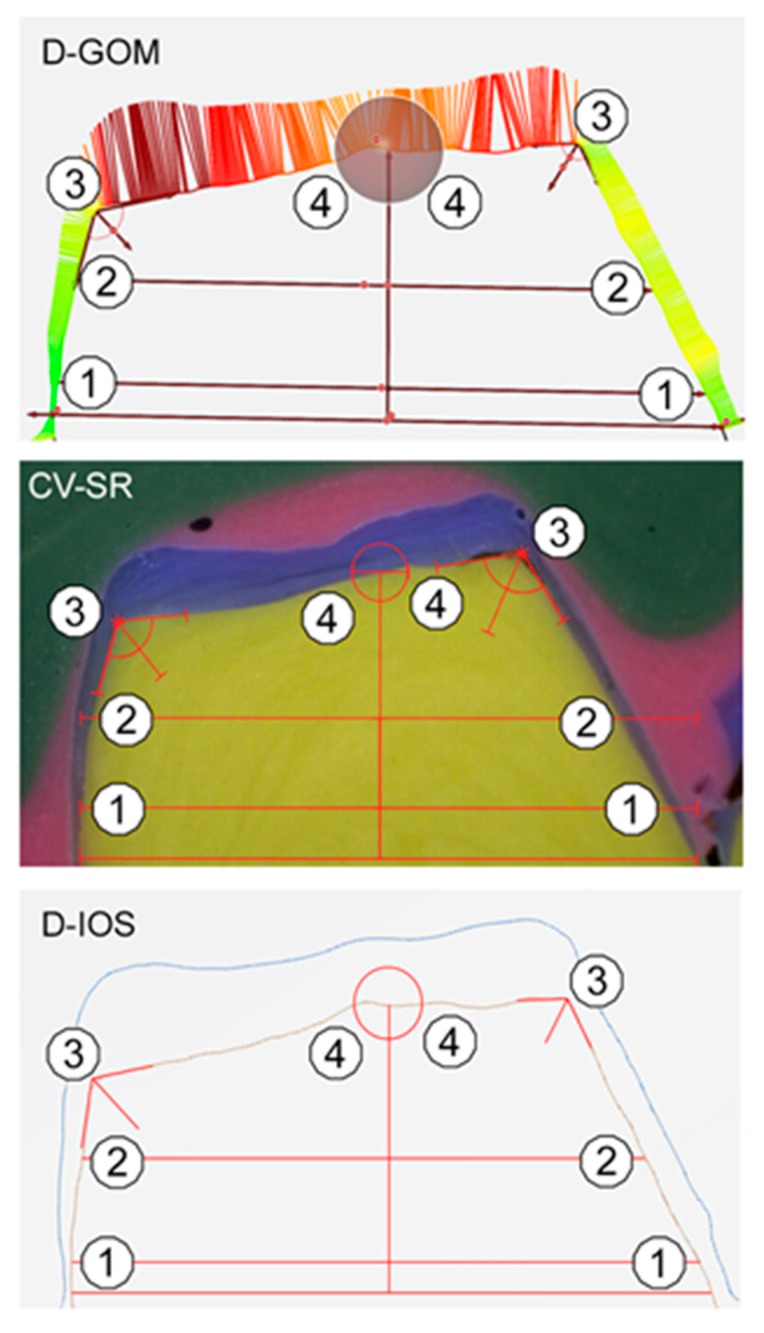
Example of the three evaluation methods (D-GOM = digital replica method with laboratory software, CV-SR = conventional silicone replica technique and D-IOS = IOS-based digital approach): 1 = marginal fit, 2 = axial Figure 3. = occlusal fit.

To ensure a standardized measurement setup for the evaluation of all three methods, each specimen was digitally (D-GOM and D-IOS) or manually sectioned (CV-SR) in the mesio-distal and oral-buccal direction, and the internal fit was determined at 16 predefined measurement points per tooth (Figure 2), and a total of 4320 measurements were performed. For standardized sectioning, the mesio-distal plane was defined in the direction of the central fissure of the adjacent teeth parallel to the axis of the prepared tooth. The oral-buccal plane was defined perpendicularly through the mesio-distal plane in the direction of the tooth axis and through the center of the prepared tooth. The silicone specimens (CV-SR) were sectioned with a razor blade, and the internal fit was analyzed with a light microscope (Smartzoom 5, PlanApo D 1.6x/ 0.1 FWD 36 mm, Zeiss, Jena, Germany) using the respective measurement software (Smartzoom 5, version 1.1, Zeiss). For further digital analysis with three-dimensional laboratory software (D-GOM), standard tessellation language (STL) data sets of the first and second digital impressions were exported from the IOS. Subsequently, both datasets were imported to three-dimensional analysis software (GOM Inspect 3D, version V8 SR1, GOM GmbH, Braunschweig, Germany) and superimposed over the adjacent teeth, using the iterative-closed-point (ICP) technique. With the D-IOS methods, both scans were automatically aligned, and the internal fit was directly analyzed with the new monitoring and measurement function of the IOS. This procedure was applied to all 30 specimens using three different coping materials and three evaluation methods. Figure 3 shows an example of the analysis of the internal fit for all three evaluation methods (CV-SR, D-GOM, D-IOS). 

Statistical analysis was performed using SPSS version 25 (IBM Corporation, Armonk, NY, USA). 2×3×3-ANOVA with factor materials and positions was calculated. Knowing that the interaction between the evaluation method and the position was very weak, only the interaction between the materials by position was interpreted. Furthermore, pairwise comparisons of the three different coping materials (COM, ZIR, NPA) were tested for every level of position (marginal, axial, or occlusal fit). The model residuals did not indicate serious deviations from a normal distribution. To consider the heterogeneity of variances, the MIXED procedure was used, and due to an alpha-error accumulation, *p*-values were corrected (Bonferroni). The level of significance was set at *p*-value < 0.05.

## 3. Results

Altogether 30 prepared teeth distributed in 10 molars, 11 premolars, and 9 incisors were investigated. Almost half of the teeth (n = 14) showed a clinical situation with mesial and distal adjacent teeth, whereas the others (n = 16) represented a terminal preparation with only mesial adjacent teeth. All 90 CAD/CAM measurement copings were feasible to investigate the internal fit of single crowns, and no manual adjustment was required. 

**Figure 4:**
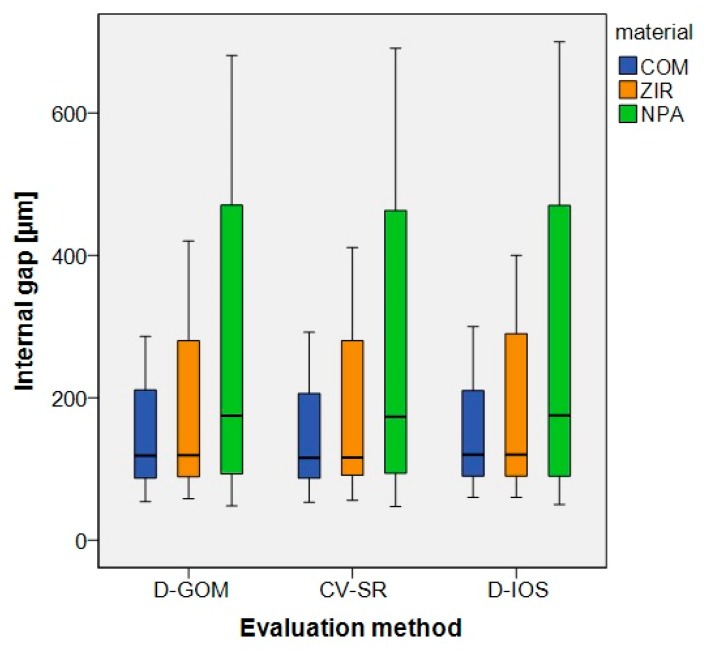
Boxplots of the three evaluation methods (D-GOM, CV-SR and D-IOS).

**Figure 5:**
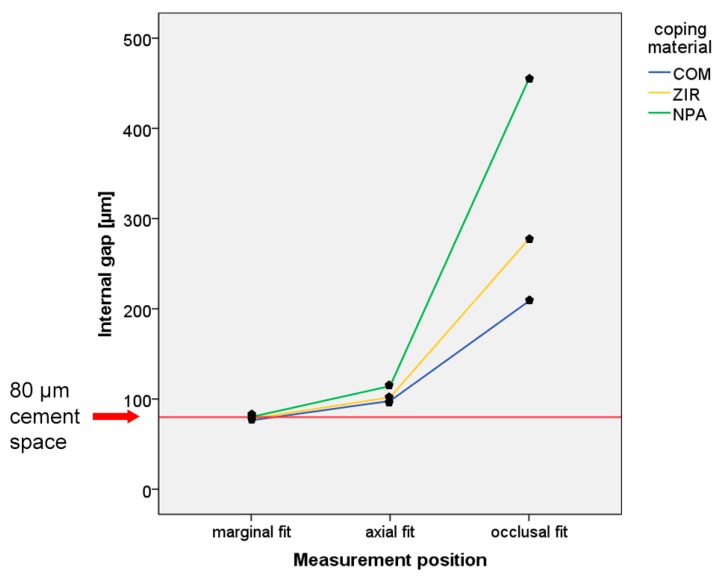
Line chart of interaction between the coping material and measurement position.

For the overall data, the results are displayed in Figure 4 and Table 2. No significant difference was observed between the three evaluation methods (CV-SR, D-GOM, D-IOS). Therefore, the first part of the null hypothesis could not be rejected. Furthermore, no significant difference was found among the tooth types and the clinical situation with mesial and distal or solely mesial adjacent teeth. However, significant differences were observed for the coping materials (*p*-value < 0.05), the single measurement position (marginal, axial, and occlusal fit) (*p*-value < 0.05), and the interaction between the coping material and the measurement position (*p*-value < 0.05) (Figure 5 and Table 3 and Table 4). Thus, the second part of the null hypothesis had to be rejected. COM showed the smallest internal gap, followed by ZIR and NPA. Regardless of the coping material, the occlusal gap was higher compared to the axial and marginal gaps. Furthermore, only the internal gaps of the marginal area almost matched the target parameter of 80-µm for cement space (Figure 5 and Table 4). This implies that the third part of the null hypothesis was partly rejected.

## 4. Discussion

All three evaluation methods (D-GOM, D-IOS, and CV-SR) were applicable for the investigation of the internal gaps in different patient situations. Even though teeth of different types (incisor, premolar, molar), sizes, and shapes were prepared manually, a standardized preparation protocol according to published guidelines was observed [31]. During the investigation, the same hardware and software of IOS and GOM Inspect were used, and no update was deployed. Only one experienced operator investigated the internal fit with all three evaluation methods to ensure standardized testing conditions. This is also a strength of this study (standardization) as well as the main weakness (operator related bias). In order to reduce this risk of bias, the evaluations were carried out at different times. 

In addition to the investigation of different evaluation methods for the analysis of internal fit, different coping materials were tested as well. The three CAD/CAM materials used in this study represent the principally used restorative material groups (polymers, ceramics, and alloys) for FDPs in dentistry today. However, most studies analyzed only one material [22,23,26,28] or compared two material groups (e.g., ceramics and polymers [37,38] or ceramics and alloys [39,40]) to each other. 

Furthermore, because Hasanzade et al. described that manual post-processing leads to a significantly higher internal gap, in this study, measurement copings were not manually adjusted [41]. In the literature, a target parameter for the cement space between 30–500 µm is described [9,22,39,42]. However, an internal gap as small as possible is recommended, but should be implementable as well. Due to the fact that the results of another laboratory study showed a better congruence between the target parameter and the actual measured internal gap for 80-µm compared to 50-µm [29], the cement space was set at 80-µm [6,43].

For the analysis of both digital evaluation methods (D-IOS and D-GOM), STL datasets were superimposed over the adjacent teeth with best-fit alignment. Mennito et al. described that superimposition over hard tissue shows significantly better alignment compared to soft tissue [44]. Zimmermann et al. [28] also used adjacent teeth for superimposition, whereas Lee [7] used a notch in the virtual die for alignment, which is not applicable for investigation in patients. O’Toole et al. [45] investigated different alignment procedures and showed significantly lower alignment errors for reference alignment compared to best-fit alignment. In the oral cavity, there is no reference structure, so some studies used additional reference aids [46,47]. However, this is not applicable for measurement in daily practice without expert skills or a complex laboratory setup.

Although the number of measurement points is not limited for both digital methods (D-GOM and D-IOS) in this study, 16 measurement points per specimen distributed to marginal, axial, and occlusal positions were selected to allow comparison with the conventional silicone replica method (CV-SR); this procedure is well described in the literature [13,20,30]. However, a comparison with other studies is often difficult because of the different setups regarding coping materials, measurement positions, and methods [4,13].

The results of this clinical study did not show a significant difference between the three evaluation methods (D-IOS, D-GOM, and CV-SR) regarding the internal fit. Rudolph et al. investigated a digital replica method with laboratory software, which is comparable to D-GOM in this study [48]. They did not find a significant difference between the conventional silicone replica technique and their digital replica method, which is in good accordance with our results. Mai et al. also compared their computer-aided replica method to the conventional silicone replica technique and did not report a significant difference [26]. 

Bosniac et al. [35] investigated the marginal fit of single crowns with caraTrios (3Shape), a preceding IOS of the Trios 3 used in this study. They described significantly higher misfit for molars compared to all other tooth types and explained the findings with limited accessibility in the posterior region due to the size of the handpiece. In this study, no significant difference in tooth type was found. In recent years, the handpieces of the Trios IOS were constructed smaller, which enables easier scanning in the oral cavity. 

Huang et al. [39] also investigated the internal fit of single crowns for different tooth types and found no significant differences. Furthermore, they showed significantly higher accuracy for non-precious alloys compared to ceramics for the marginal and axial measurement positions. These findings are in contrast to those of Pimienta et al. [1] and the results of our study, where NPA showed the highest internal gap compared to ZIR and COM. However, regarding occlusal positions, Huang et al. [39] also described significantly higher internal gaps for non-precious alloys, which agrees with our findings. 

Rezende et al. described a significant discrepancy between the target parameter of the cement space and the actual measured internal gap [6]. In this study, only the internal fit in the marginal measurement position almost matched the predefined 80-µm. This might be explained by the milling radius correction of the CAM strategy. Zimmermann et al. showed a significant difference in the accuracy of fit for ceramic crowns milled by different CAM strategies [49]. Today, digital preparation analysis (e.g., prepCheck, Dentsply Sirona) enables chairside control of the preparation and a real-time correction. Future software developments may also display the required milling radius for better internal fit of CAD/CAM-fabricated restorations. This can improve the internal fit of CAD/CAM restorations and reduce failure. Furthermore, new CAD/CAM materials and workflows (e.g., polyaryletherketones (PAEKs), 3D printing) will be available in the near future [11,12], which require an adjustment of milling or printing parameters. The chairside control of the internal fit gives dentists an easily applicable tool for their own quality management and development of new workflows. 

The chairside analysis of the internal fit with D-IOS allows a feasible inspection of the restoration in the dental office without expert skills or laboratory equipment. This study showed that the evaluation method could be applied to different CAD/CAM materials. Overall, further studies should investigate the applicability of D-IOS in various dental offices with different operators. Furthermore, only single crown preparations were analyzed, but the digital analysis of inlays, partial crowns, and bridges is conceivable as well. In the future, improvements of the current IOS systems should include automatic matching and artificial intelligence features to analyze the internal fit. This would decisively help to accelerate the evaluation process and extend its application in dental offices.

## 5. Conclusions

The new intraoral scanner-based chairside measurement method is applicable for the analysis of the internal fit of single crowns in different clinical settings and did not show significant differences compared to the conventional silicone replica method and the digital replica method with laboratory software.

## Figures and Tables

**Table 1:** Materials used in this study (information provided by the manufacturer).

Material	Product Name (Batch No.)	Manufacturer
Powder spray	CEREC Optispray (S50868)	Dentsply Sirona (Bensheim, Germany)
Low-viscosity addition-curing silicone	Fit Test C&B (1841465)	Voco (Cuxhaven, Germany)
Low-viscosity vinyl polyether silicone	EXA’lence Extra Light Body (1806261)	GC (Tokyo, Japan)
Low-viscosity vinyl polyether silicone	EXA’lence Light Body (1806221)	
High-viscosity vinyl polyether silicone	EXA’lence Putty (1711091)	
Resin composite block	LuxaCam Composite (784249)	DMG (Hamburg, Germany)
3Y-TZP zirconium dioxide	Lava Plus (4525742)	3M (St.Paul, MN, USA)
Non-precious alloy (>10 wt% cobalt, chrome, 1–10 wt% tungsten, silicon, manganese, iron, 0.1–1 wt% carbon)	Finoframe CoCr (K10627)	Fino (Bad Bocklet, Germany)

**Table 2:** Descriptive statistics of the internal gap [µm] for the three evaluation methods (CV-SR, D-GOM and D-IOS), coping material (COM = resin composite, ZIR = zirconium dioxide and NPA = non-precious alloy) and measurement position (marginal, axial and occlusal fit).

Evaluation Method	Coping Material	Measurement Position	Internal Gap [µm]				
			Mean	Standard Deviation	Median	Confidence Interval	
						Lower	Upper
CV-SR	COM	marginal fit	75.8	9.9	77.0	74.0	77.6
		axial fit	96.4	9.3	96.0	94.7	98.1
		occlusal fit	204.9	39.5	206.0	199.9	209.9
	ZIR	marginal fit	77.8	9.2	77.0	76.2	79.5
		axial fit	100.2	9.3	100.5	98.5	101.8
		occlusal fit	273.9	59.9	280.0	266.3	281.5
	NPA	marginal fit	79.8	12.6	79.0	77.5	82.1
		axial fit	112.5	21.1	106.0	108.7	116.3
		occlusal fit	451.1	147.6	463.0	432.4	469.9
D-GOM	COM	marginal fit	76.6	9.5	77.0	74.9	78.4
		axial fit	98.0	10.8	96.5	96.0	100.0
		occlusal fit	208.2	38.3	211.0	203.3	213.0
	ZIR	marginal fit	77.9	8.8	78.0	76.3	79.5
		axial fit	101.6	9.6	101.0	99.9	103.4
		occlusal fit	277.3	59.0	280.0	269.8	284.8
	NPA	marginal fit	80.1	11.7	80.5	78.0	82.2
		axial fit	113.9	20.6	107.0	110.2	117.6
		occlusal fit	455.2	146.9	470.5	436.6	473.9
D-IOS	COM	marginal fit	76.7	10.1	80.0	74.8	78.5
		axial fit	99.1	10.8	100.0	97.1	101.0
		occlusal fit	212.4	39.5	210.0	207.4	217.4
	ZIR	marginal fit	78.7	9.2	80.0	77.0	80.3
		axial fit	103.8	10.2	100.0	101.9	105.6
		occlusal fit	281.5	59.5	290.0	274.0	289.1
	NPA	marginal fit	80.4	12.3	80.0	78.2	82.6
		axial fit	116.4	20.6	110.0	112.7	120.1
		occlusal fit	458.7	147.2	470.0	440.0	477.4

**Table 3:** *P*-values of the internal gap pairwise comparison (*p*-value < 0.05, COM = resin composite, ZIR = zirconium dioxide and NPA = non-precious alloy).

Measurement Position		ZIR	NPA
marginal fit	**COM**	*p* = 0.037	*p* < 0.001
	**ZIR**		*p* = 0.040
axial fit	**COM**	*p* < 0.001	*p* < 0.001
	**ZIR**		*p* < 0.001
occlusal fit	**COM**	*p* < 0.001	*p* < 0.001
	****ZIR****		*p* < 0.001

**Table 4:** Results of internal fit measurement (pooled data for the three evaluation methods, COM = resin composite, ZIR = zirconium dioxide and NPA = non-precious alloy).

Measurement Position	Internal Gap (mean (SD) [µm])			Internal Gap – Cement Space = Absolute Discrepancy (Mean (SD) [µm])		
	COM	ZIR	NPA	COM	ZIR	NPA
marginal fit (n = 120)	76.4 (0.5)	78.1 (0.5)	80.1 (0.6)	−3.6 (0.5)	−1.9 (0.5)	0.1 (0.6)
axial fit (n = 120)	97.8 (0.6)	101.8 (0.5)	114.3 (1.1)	17.8 (0.6)	21.8 (0.5)	34.3 (1.1)
occlusal fit (n = 240)	208.5 (1.5)	277.6 (2.2)	455.0 (5.5)	128.5 (1.5)	197.6 (2.2)	375.0 (5.5)
